# Physical, biochemical, and biological characterization of olive-derived lipid nanovesicles for drug delivery applications

**DOI:** 10.1186/s12951-024-02964-w

**Published:** 2024-11-18

**Authors:** Zhu Zhao, Jerome Lacombe, Laurianne Simon, Noelia M. Sanchez-Ballester, Ashkan Khanishayan, Naina Shaik, Kallie Case, Pierre-Yves Dugas, Mathieu Repellin, Giovanna Lollo, Ian Soulairol, Ashlee F. Harris, Michael Gordon, Sylvie Begu, Frederic Zenhausern

**Affiliations:** 1 Center for Applied NanoBioscience and Medicine, College of Medicine Phoenix, University of Arizona, Phoenix, AZ 85004 USA; 2 Department of Biomedical Engineering, College of Engineering, The University of Arizona, Tucson, AZ 85721 USA; 3 Department of Basic Medical Sciences, College of Medicine Phoenix, University of Arizona, Phoenix, AZ 85004 USA; 4 ICGM, CNRS, ENSCM, University of Montpellier, Montpellier, 34000 France; 5 University of Lyon, Université Claude Bernard Lyon 1, CNRS, CP2M UMR 5128, Villeurbanne, France; 6 University of Lyon, Université Claude Bernard Lyon 1, CNRS, LAGEPP UMR 5007, 43 bd 11 Novembre 1918, Villeurbanne, 69622 France; 7 Institut universitaire de France (IUF), Paris, France; 8 Department of Pharmacy, Nîmes University Hospital, Nîmes, 30900 France; 9 HonorHealth Research Institute, Scottsdale, AZ 85258 USA

## Abstract

Extracellular vesicles (EVs) have shown great promise as drug delivery system (DDS). However, their complex and costly production limit their development for clinical use. Interestingly, the plant kingdom can also produce EV-like nanovesicles that can easily be isolated and purified from a large quantity of raw material at a high yield. In this study, olive-derived nanovesicles (ODNVs) were isolated from raw fruits using serial centrifugations and their physical and biological features characterized to demonstrate their promising potential to be used as a DDS. Nanotracking particle analysis indicated an average size of 109.5 ± 3.0 nm and yield of 10^12^ ODNVs/mL for the purest fraction. Microscopy imaging, membrane fluidity assay and lipidomics analysis showed the presence of a rich lipid bilayer that significantly varied between different sources of ODNVs but showed a distinct signature compared to human EVs. Moreover, ODNVs were enriched in PEN1 and TET8 compared to raw fruits, suggesting an extracellular origin. Interestingly, ODNVs size and yield stayed unchanged after exposure to high temperature (70 °C for 1 h), wide pH range (5–10), and 50–100 nm extrusion, demonstrating high resistance to physical and chemical stresses. This high resistance allowed ODNVs to stay stable in water at 4 °C for a month, or with the addition of 25 mM trehalose for long-term freezing storage. Finally, ODNVs were internalized by both 2D and 3D cell culture without triggering significant cytotoxicity and immunogenicity. Importantly, the anticancer drug doxorubicin (dox) could be loaded by passive incubation within ODNVs and dox-loaded ODNVs decreased cell viability by 90% compared to only 70% for free dox at the same concentration, indicating a higher efficiency of drug delivery by ODNVs. In addition, this high cytotoxicity effect of dox-loaded ODNVs was shown to be stable after a 2-week storage at 4 °C. Together, these findings suggested that ODNVs represent a promising candidate as drug nanocarrier for various DDS clinical applications, as demonstrated by their biocompatibility, high resistance to stress, good stability in harsh environment, and improvement of anticancer drug efficacy.

**Supplementary Information:**

The online version contains supplementary material available at 10.1186/s12951-024-02964-w.

## Introduction

Drug delivery systems (DDS) have been shown to improve treatment outcomes by decreasing toxicity, improving efficacy, enhancing patient compliance and enabling new unprecedented therapeutic approaches [1]. Nanotechnology has contributed substantially to the development of drug carriers, and over decades, various synthetic nanoparticle DDS have emerged. These have been utilized to improve the pharmacokinetic and pharmacodynamic of different classes of therapeutics [2, 3]. However, the clinical applications of synthetic nanoparticles remain difficult due to the immune intolerance or the presence of physical and biological barriers, decreasing stability and efficiency [4]. To overcome these limitations, natural carriers have gained attention, such as extracellular vesicles (EVs) [5]. EVs are a heterogeneous population of nanosized bilayer-enclosed vesicles secreted from all cell types (i.e., animal and vegetal) that carry DNA, RNA, proteins and lipids, facilitating cell-to-cell communication [6, 7]. Several EVs properties open the door to serve as a drug delivery vehicle, including their intrinsic stability in circulation (negatively charged surface), their ability to avoid immune system (presence of surface biomarkers), and their capacity to cross biological barriers by exploiting endogenous intracellular trafficking mechanisms or by displaying inherent targeting properties [8]. However, their development is still limited by challenges in manufacturing to produce large quantities at low cost and high quality, limited drug loading efficiency or low resistance to environmental factors [9].

Interestingly, recent evidence has identified the presence of exosome-like vesicles in the apoplastic fluid of plants, suggesting that the vegetal kingdom also produces EVs [10–13]. Such EVs can be isolated directly from the apoplast or after tissue disruption where a recent nomenclature proposed to name them plant-derived nanovesicles (PDNVs) [14]. PDNVs share structural similarities with animal-derived EVs, including their nanosized structure and double-layered membrane, which effectively mediate the intercellular transfer of bioactive components [15] and can even successfully deliver exogenous and endogenous agents to animal cells [16]. Interestingly, PDNVs also showed low cytotoxicity and immunogenicity in vivo with no induction of pro-inflammatory cytokines or liver enzymes compared to liposome treatment [17]. Depending on the source, PDNVs even demonstrated anti-inflammatory effects by decreasing the secretion of pro-inflammatory cytokines [18], inhibiting reactive oxygen species production or by blocking inflammation signaling pathway [19].

Therefore, PDNVs have recently emerged as a potential candidate DDS with the promise of counteracting the main drawbacks of animal EVs such as high production cost and low environmental resistance reducing their shelf-life and their bioengineered ability [20]. Thus, many studies have shown the great potential of PDNVs for biomedical use, either for their natural therapeutic properties or their ability to carry and release drug to the cellular target [21, 22]. Because of their biocompatibility and availability, edible plants have been the main focus of these works, including citrus fruits [23–26], bitter melon [27], apple [28], cabbage [29], ginger [30–34], garlic [35], turmeric [36], grapefruit [17, 37–41], broccoli [42, 43], barbado cherry [44], tomato [45–47], grape [48], mushroom [49], etc. Despite the growth on PDNVs field, there is still a limited understanding of their biological properties, drug loading and delivery performance, physical stability, and preservation methods [50].

Olives are one of the most extensively cultivated and fast expanding fruit crops in the world [51]. Olive fruit’s average composition includes water (50%), protein (1.6%), oil (22%), carbohydrate (19.1%), cellulose (5.8%), inorganic substances (1.5%) and phenolic compounds (1–3%) [52]. The latter, that mostly consist of flavanols, phenolic acids and derivatives, secoiridoids and phenolic alcohols, have shown many biological activities for health benefits including antioxidant, anti-inflammatory, antimicrobial, anti-tumor, or anti-hypertensive properties [52]. However, to our knowledge, no studies have investigated whether nanovesicles could be isolated from olive fruits, and only one work reported the presence of nanovesicles in the waste generated during olive oil production [41]. Through an extensive physical, biochemical and biological characterization, this study aimed to demonstrate that nanovesicles can be isolated from olive fruits and to evaluate if these olive-derived nanovesicles (ODNVs) have the required properties to serve as a new DDS.

## Materials and methods

### Reagents

1,6-diphenyl-1,3,5-hexatrience (DPH) was brought from Fluka (France). Hydrochloric acid solution at 37%, sodium hydroxide, tetrahydrofuran (THF), sodium carbonate (Na_2_CO_3_, 223484), Tween20 (P1379), 3,3’,5,5’-Tetramethylbenzidine (TMB) Liquid Substrate (T4444), Stop Reagent for TMB Substrate (S5814), PKH67 Green Fluorescent Cell Linker Kit (#PKH67GL-1KT), doxorubicin hydrochloride (dox) (D1515), Lipopolysaccharides from Escherichia coli O111:B4 (LPS, L2630) were purchased from Sigma Aldrich. Fetal Bovine Serum (FBS, 16140071), Exosome-depleted Fetal Bovine Serum (A2720803), Penicillin-Streptomycin (10,000 U/mL) (15140122), Pierce™ BCA Protein Assay Kit (#23225), Alexa Fluor^™^ 555 Phalloidin (A34055), Dulbecco’s phosphate-buffered saline (DPBS) (Gibco, 14190144), RIPA Lysis and Extraction Buffer (89900), Paraformaldehyde Solution, 4% in PBS (J19943.K2) and Total Exosome Isolation Reagent (from cell culture medium) (4478359) were purchased from ThermoFisher Scientific. Rabbit anti-syntaxin-121 (PEN1) (PHY2912S) and anti-tetraspanin-8 (TET8) (PHY1491S) antibodies were purchased from PhytoAB. Horseradish peroxidase–conjugated polyclonal anti-rabbit IgG (111-035-003) was purchased from Jackson ImmunoResearch. Tissue-Tek* O.C.T. Compound (Sakura Finetek, 4583) was purchased through VWR. Sodium Bicarbonate (NaHCO3, BP328) was purchased from Fisher Scientific.

### Cell lines

Human A549 lung adenocarcinoma (CCL-185), SKOV3 ovarian cancer (HTB-77) and HULEC-5a microvascular endothelial (CRL-3244) cell lines were purchased from The American Type Culture Collection. A549 and SKOV3 cells were cultured in Kaighn’s Modification of Ham’s F-12 Medium and McCoy’s 5 A Medium, respectively. Both media were supplemented with 10% FBS and 1% penicillin/streptomycin. HULEC-5a were cultured in MCDB 131 Medium, supplemented with 10% FBS, 10mM Glutamine, 1% penicillin/streptomycin, 10 ng/mL human epidermal growth factor and 1 µg/mL hydrocortisone. All cell lines were maintained in a humidified incubator (37 °C, 5% CO_2_).

### Isolation and purification of olive-derived NanoVesicles (ODNVs)

Canned black olives were purchased at the local grocery store and fresh, unprocessed olives were harvested from local orchard. Olives were first rinsed with deionized (DI) water and weighted to 100 g. Fifty milliliters of DI water were then added and the mixture was blended using a kitchen blender until the obtention of a smoothy-like solution. The blended solution was differentially centrifuged at 4 °C (4 800 g for 25 min, 6 000 g for 25 min (x2) and 10 000 g for 1 h). The supernatant of the final 10 000 g spin was then centrifuged at 100 000 g for 2 h at 4 °C to collect a pellet containing nanovesicles that was resuspended in 1 mL of DI water and referred to as Whole Fraction (WF). The WF was then transferred on top of a sucrose step gradient (8/15/30/45/60 w/v%) and centrifuged at 100 000 g for 2 h at 4 °C. The bands between each sucrose layer were harvested and named as F1, F2, F3, F4 and F5. Each fraction was then mixed with 10 mL of DI water and centrifuged at 100 000 g for 2 h at 4 °C to remove excess sucrose. After this final centrifugation, the supernatant was discarded, and each fraction was resuspended into 100 µL of DI water and stored at 4 °C until use. The workflow of this production and isolation process is represented in Supplementary Fig. 1.

### Isolation and purification of A549 EVs

For lipidomic analysis and cytokine assay, A549 EVs were also isolated. A549 were cultured in T-75 flasks in complete medium until reaching 60% confluency. Complete medium was then replaced by exosome-depleted serum-containing medium and cells were cultured for 48 h. Supernatant was collected and centrifuged at 2 000 g for 30 min at 4 °C to remove cell debris. The cell-free supernatant was then transferred into a new tube and the Total Exosome Isolation (from the cell culture medium) reagent was added at a ratio of 2:1. The mixture was then incubated at 4 °C overnight. After incubation, the mixture was centrifuged at 10 000 g for 1 h at 4 °C. The supernatant was discarded, and the pellet was resuspended in 100 µL of DPBS. The mode size and concentration of A549 EVs were determined by NTA (Supplementary Fig. 2).

### Particle size and surface charge analysis

The size distribution and concentration of ODNVs were assessed by nanoparticle tracking analysis (NTA) using a NanoSight NS300 (Malvern Panalytical, UK). Per manufacturer’s recommendations, the ODNVs were diluted with degassed DI water before injection into the flow cell. Zeta potential measurements were conducted with Zetasizer NanoZS apparatus (Malvern Panalytical, UK). ODNVs solution was prepared at a concentration of 2.10^9^ particles/mL in DPBS at 1 mM and placed in folded capillary zeta cell (Malvern). Polydispersity Index (PDI) was calculated using the equation:

$$\:PDI=\:{\left(\frac{\sigma\:}{d}\right)}^{2}$$

where σ is the standard deviation of the ODNVs population and d the ODNVs mean diameter.

### Cryogenic electron microscopy (Cryo-EM)

Three microliters of ODNVs solution at a concentration of 1.10^12^ particles/mL was deposited onto a 300 mesh Lacey carbon film grid (EMS) and quench-frozen in liquid ethane using a Thermo Fisher Vitrobot. This procedure produced a protective vitreous ice to maintain sample integrity throughout imaging time. The cryo-EM images were taken using a cryo-electron microscope JEOL 1400 F − 120 kV LaB6 (Centre Technologique des Micro¬structures (CTµ) – University Claude Bernard Lyon 1, Villeurbanne, France) using a precooled cryo-transfer holder (Fischione 2550) and a Gatan Rio16 camera used in low-dose conditions.

### Fluorescence anisotropy measurement for membrane fluidity assessment

A stock solution of DPH was prepared at 0.947 mM in THF. ODNVs solution at a concentration of 1.10^12^ particles/mL was stained with 10 µL of the DPH stock solution in pure water. The mixture was placed in dark for 1 h at 60 °C under stirring at 200 rpm.

Anisotropy measurements were performed using a spectrofluorometer RF6000 (Shimadzu, Japan) equipped with polarizers. Samples were excited at 360 nm and the fluorescence intensity was determined at 430 nm with a temperature ramp from 20 to 76 °C at 2 °C/min using temperature controller TC1 (Quantum Northwest, USA). The anisotropy (r) was calculated using the equation:

$$\:r=\frac{Ip-G\text{*}Ipp}{Ip+2\text{*}G\text{*}Ipp}$$

where Ip and Ipp are the intensities measured with parallel and perpendicular polarizers, respectively. The G factor was determined at 0.81.

### Enzyme linked immunoSorbent assay (ELISA)

ELISA was used to detect and quantify TET8 and PEN1 proteins in *Arabidopsis Thaliana* (*A. Thaliana*) stems and leaves, olive fruits and nanovesicles derived from these tissues. The WF of *A. Thaliana-*derived nanovesicles (WF ADNVs) was isolated by the same method of the WF ODNVs, as described above. Proteins from *A. Thaliana* stems and leaves and olive fruits were extracted as follows: 20 mg of *A. Thaliana* and olives were frozen by liquid nitrogen and grinded into powder. The powder was then lysed with 100 µL RIPA buffer on ice for 15 min under agitation. The lysed solution was then centrifuged at 14 000 g for 20 min at 4 °C and the supernatant collected and stored at -80 °C until use. The protein extracts from *A. Thaliana*, olive fruits, WF ADNVS, WF ODNVs and F3 ODNVs were quantified by BCA assay. Proteins were then resuspended in filtered ELISA coating buffer (15 mM Na_2_CO_3_, 35 mM NaHCO_3_, pH 9.6) at a concentration of 10 µg/mL and coated on MaxiSorp High Protein-Binding (Nunc) plates at 4 °C overnight. The coated wells were then washed three times with 1X PBS + 0.5% Tween20 (PBST) and blocked with PBST + 0.6% non-fat dry milk 1 h at 37 °C. After blocking, anti-TET8 (1/1000) or anti-PEN1 (1/1000) antibodies diluted in PBST + 0.6% non-fat dry milk + 0.8 M NaCl were added to the well for 1 h at room temperature under agitation. After 3 washes with PBST, the plates were incubated with a horseradish peroxidase–conjugated polyclonal anti-rabbit (1:5000) for 1 h at room temperature under agitation. After 3 washes with PBST, the plates were incubated with TMB substrate solution for 15 min, and absorbance values were read at 450 nm using a microplate spectrophotometer BioTek Epoch (Agilent, CA) after addition of the stop solution.

### Lipidomics analysis

Lipids were extracted from ODNVs and A549 EVs following a modified Folch protocol. ODNVs and A549 EVs were mixed with 4 mL cold methyl tert-butyl ether and 2 mL cold methanol in a glass tube. After 60 s vortex, 1.5 mL ice-cold Milli-Q water was added to induce phase separation. The samples were vortexed for 60 s and incubated at 4 °C for 1 h. The organic phase containing lipids was then removed, transferred into a new tube and dried down using nitrogen gas. The extract was then redissolved in 60:40 of A: B solvents for LC-MS analysis.

Metabolite extracts were analyzed using liquid chromatography electrospray ionization tandem mass spectrometry (LC-ESI-MS/MS) using the Vanquish UHPLC system (ThermoFisher Scientific, USA) interfaced with the Q-Exactive Orbitrap mass spectrometer (ThermoFisher Scientific, USA).

#### RP LC parameters

One microliter of extract was injected and compounds separated using a Waters ACQUITY Premier HSS T3 column (1.8 μm, 2.1 mm x 150 mm) with a column temperature of 45 °C and a flow rate of 250 µL/min under the following conditions: mobile phase A (50:50 of acetonitrile: H_2_O with 10 mM ammonium acetate and 0.1% formic acid) and B (88:10:2 of isopropanol: acetonitrile: H_2_O with 10 mM ammonium acetate and 0.1% formic acid). The gradient method was from: 40–50% B 0–2 min, 50–60% B 2–3 min, 60–70% B 3–12 min, 70–75% B 12–15 min, 75–78% B 15–17 min, 78–85% B 17–19 min, 85–92% B 19–22 min, 92–99% B 22–25 min, hold 99% B 25–34 min.

#### MS parameters

Samples were analyzed in positive ionization mode using higher-energy collision dissociation. The HESI source parameters were set as follows: spray voltage 3.5 kV; capillary temperature 300 °C; S lens RF level 50 arbitrary units, and aux gas heater temperature 300 °C. Full MS scan data were acquired at a resolving power of 70 000 FWHM at m/z 200 with the scanning range of m/z 150–1500. The automatic gain control (AGC) target is set at 3E6, with the maximum injection time of 100 ms. The data dependent acquisition (dd-MS2) parameters used to obtain product ion spectra are as follows: resolving power 17 500 FWHM at m/z 200, AGC target of 1E5 with maximum injection time was set to 50 ms, isolation width 4.0 m/z, and HCD collision energies of: 25, 30 eV.

#### Identifications/relative quantification

For data generated, confident metabolite identifications were made using Thermo Lipid Search 5.0 software. Spectra were aligned using a maximum of 0.5 min RT shift, a 5 ppm mass tolerance, and S/N threshold of 3. Peaks were selected based on a minimum intensity of 3E5 and a chromatographic S/N of 3. Detected features were grouped based on a mass tolerance of 5 ppm and a RT tolerance of 0.5. Compounds were assigned based on Isotopic pattern, MS1, and MS2. Non-normalized values for peak areas were then exported to Excel (Microsoft Corp., Redmond, WA, USA) for quantile normalization and z-score calculation. Principal component analysis (PCA) and hierarchical clustering were employed to classify and discriminate the samples according to the multidimensional data generated from the analytical techniques, and visualized with SRplot [53] and Morpheus (https://software.broadinstitute.org/morpheus).

Identified lipid class were: FAs, fatty acyls; GLs, glycerolipids; GPs, glycerophospholipids; Prenol lipids; Sphs, sphyngolipids; Sterol lipids; and subclasses were AcCa, fatty acyls carnitines; AcHexChE, acylhexosyl cholesterol ester; AcHexCmE, acylhexosyl campesterol ester; AcHexSiE, acylhexosyl sitosterol ester; AcHexZyE, acylhexosyl zymosterol ester; AEA, N-acyl ethanolamines; BisMePA, bis-methyl phosphatidic acid; Cer, ceramide; CerP, ceramide phosphate; CerPE, ceramide phosphoethanolamine; Ch, cholesterol; ChE, cholesterol ester; Co, coenzyme Q; DG, diglyceride; DGDG, digalactosyldiacylglycerol; Hex1Cer, ceramide monosaccharides; Hex2Cer, ceramide disaccharides; LBPA, lysobisphosphatidic acid; LPA, lysophosphatidic acid; LPC, lysophosphatidylcholine; LPE, lysophosphatidylethanolamine; LPEt, lysophosphatidylethanol; LPG, lysophosphatidylglycerol; LPI, lysophosphatidylinositol; LPS, lysophosphatidylserine; MePC, methyl phosphatidylcholine; MG, monoglyceride; MGDG, digalactosylmonoacylglycerol; PA, phosphatidic acid; PC, phosphatidylcholine; PE, phosphatidylethanolamine; PEt, phosphatidylethanol; PG, phosphatidylglycerol; PI, phosphatidylinositol; PIP2, phosphatidylinositol bisphosphate; PS, phosphatidylserine; SiE, sitosterol ester; SM, sphingomyelin; SPH, sphingosine; SPHG1, sphingosine 1-phosphate; ST, sulfoglycosphingolipid; TG, triglyceride; WE, wax ester.

### ODNVs cellular toxicity

For 2D culture, A549 and SKOV3 cells were seeded at a density of 4000 cells/well in a tissue culture treated 96-well plate and allowed to attach overnight. Cells were then exposed to ODNVs at concentration of 0, 10^6^, 10^7^, 10^8^, 10^9^, 10^10^ and 10^11^ particles/mL. After 3 days of incubation, cell viability was assessed by modified MTT assay according to the manufacturer’s recommendations (CellTiter 96 Non-Radioactive Cell Proliferation Assay, Promega, WI). Absorbance was recorded at 570 nm using microplate spectrophotometer BioTek Epoch (Agilent, CA).

For 3D culture, A549 and SKOV3 cells were seeded at a density of 4000 cells/well in 96-well Ultra-low Attachment Plates (MS-9096UZ, S-bio). After 4 days, spheroids were exposed to ODNVs at concentration of 0, 10^6^, 10^7^, 10^8^, 10^9^, 10^10^ and 10^11^ particles/mL. Spheroids were imaged under bright field microscopy every 2 days for 8 days and their long (D) and short (d) diameters measured using ImageJ. Spheroid volume (V) was then calculated using the formula:

$$\:V=\:\frac{4}{3}\pi\:{\left(\frac{D+d}{4}\right)}^{3}$$

### ODNVs cytokine assay

HULEC-5a cells were seeded at a cell density of 100 000 cells/well in a tissue culture-treated 12 well plate and allowed to attach overnight. Cells were exposed to 1.10^9^ A549 EVs/mL, 10^9^ ODNVs/mL and 1 µg/mL LPS for 24 h. The supernatant was then collected, centrifuged at 4000 g for 10 min to remove any visible particulate material and stored at −80 °C until use. The level of 8 different pro-inflammatory cytokines (IL-1α, IL-1β, IL-6, IL-8, GM-CSF, INF-γ, MCAF and TNF-α) was then assessed using the Multiplex Human Cytokine ELISA Kit (MBS590064, MyBiosource) following the manufacturer’s instructions.

### ODNVs labelling and cellular internalization

ODNVs were labeled with the PKH67 Green Fluorescent Cell Linker Kit following the manufacturer’s protocol with minor modifications. Briefly, 2 µL of PKH67 dye was added to 0.5 mL of Diluent C. This mixture was then incubated with 100 µL of ODNVs solution or 100 µL of PBS (negative control) in 0.5 mL of Diluent C for 10 min. After incubation, the mixture was filtrated by ultrafiltration with a 100-kDa filter (Amicon Ultrafilter, Millipore, MA) to remove free PKH67 dye using 3 rounds of centrifugation at 4 000 g for 10 min.

For 2D culture, the A549 and SKOV3 cells were seeded on tissue culture treated coverslip at a concentration of 10,000 cells/mL and cultured with PKH67-labeled ODNVs at a concentration of 1.10^12^ particles/mL or PBS (negative control) overnight. After incubation, the cells were fixed with 4% paraformaldehyde for 15 min and counterstained with Alexa Fluor™ 555 Phalloidin and DAPI.

For 3D culture, A549 and SKOV3 cell lines were seeded at a density of 4000 cells/well in 96-well Ultra-low Attachment Plates (MS-9096UZ, S-bio). After 4 days, spheroids were cultured with PKH67-labeled ODNVs at 1.10^12^ particles/mL or PBS (negative control) for 3 days. After incubation, spheroids were frozen in OCT solution and cut in 15 μm slices by a CM195 cryostat (Leica, US).

The images were obtained using a Zeiss Axio Imager M2 epifluorescent microscope (Zeiss, Germany), acquired with a Zeiss AxioCam MRm camera using ZEN 4.5 software and analyzed with ImageJ. For 2D culture, corrected fluorescence intensity of each frame (CFIF) was calculated based on the following formula: CFIF = Frame Integrated Density – (Area of selected frame x Mean fluorescence of background readings) and reported as fluorescence intensity per cell by dividing CFIF by the number of cells in each frame. For 3D culture, fluorescence intensity signal of each spheroid was calculated following the formula: Spheroid Integrated Density / Spheroid Area.

### ODNVs stability tests to external stresses

#### Resistance to temperature

ODNVs solution at a concentration of 1.10^9^ particles/mL was incubated for 1 h at room temperature (25 °C), 50 °C, 60–70 °C. Mode size and concentration of each condition were analyzed by NTA.

#### Resistance to salt concentration

ODNVs solutions were prepared in DPBS at 150, 100, 50, 10, 5, 1 mM with dilution in pure water (MilliQ water from Merck Millipore). For each solution, the concentration of ODNVs was fixed at 1.10^9^ particles/mL. The samples were incubated for 24 h at 4 °C. Mode size and concentration were analyzed by NTA.

#### Resistance to pH

Buffers solutions were prepared in pure water at pH 2.3 with gradual addition of hydrochloric acid of 0.1 M and at pH 5 with a solution of hydrochloric acid of 0.01 M. The buffer at pH 10 was made using a solution of sodium hydroxide at 0.1 M. For each solution, the concentration of ODNVs was fixed at 1.10^9^ particles/mL. The samples were incubated for 24 h at 4 °C. Mode size and concentration were analyzed by NTA.

#### Resistance to deformability with extrusion

ODNVs solution at a concentration of 1.10^9^ particles/mL were extruded at room temperature using a syringe-based hand-held mini-extruder LiposoFast-Basic (Avestin, Canada) through a 100 nm pore size polycarbonate membrane (Avestin Europe GmbH, Germany) 10, 15 and 20 times or through a 50 nm pore size membrane 5, 10 and 15 times. The resulting solutions were analyzed by NTA to determine mode size and concentration.

#### Stability performance in serum

ODNVs solution at a concentration of 1.10^9^ particles/mL were incubated in 50% exosome-depleted serum at 37 °C for 1 h and 24 h. Mode size and concentration were analyzed by NTA.

For each external stress condition, results were expressed as recovery yield (%) using the following equation:

$$\:recovery\:yield\:\left(\%\right)=\frac{concentration\:post-exposure}{concentration\:pre-exposure}*100$$

### Stability tests of different storage conditions

ODNVs at a concentration of 1.10^9^ particles/mL was resuspended in DI H_2_O, DI H_2_O with 25 mM Trehalose, 1X DPBS and 1X DPBS with 25 mM Trehalose. Each solution was then stored at 4 °C in a fridge, 25 °C on benchtop, −80 °C in freezer or lyophilized. For lyophilization, samples were first frozen by liquid nitrogen before being placed in a FreeZone 4.5 L freeze dryer (Labonco, US). The frozen samples and lyophilized samples were stored for 1 day before being thawed and reconstituted in DI H_2_O at 4 °C. After recovery, all samples were stored for 1, 7 or 14 days. After each timepoint, size of mode and concentration of each sample were analyzed by NTA.

### Doxorubicin loading and delivery by ODNVs

To evaluate the cargo capacity of ODNVs to carry and deliver drug, 100 µL of the anti-cancer drug doxorubicin (dox) at a concentration of 200 µM was incubated with 100 µL of ODNVs at a concentration of 1.10^12^ particles/mL (or 100 µL DPBS as vehicle control) and loaded following different methods, including: 1 or 2 cycles of freeze-thaw (−80 °C for 20 min / 25 °C for 10 min); 5, 20 and 30 min of water bath sonication (35 kHz); and passive incubation at 37 °C for 4 h. After loading, the dox-ODNVs solution was filtrated by ultrafiltration with a 100-kDa filter (Amicon Ultrafilter, Millipore) to remove free unloaded dox using 3 rounds of centrifugation at 4000 g for 10 min. Dox concentration was determined using its intrinsic fluorescence due to its central anthracycline chromophore group (470 nm Ex / 595 nm Em) [54]. First, fluorescence intensity of unloaded ODNVs and different concentrations of free dox (100, 50, 25, 12.5, 6.25 and 3.125 µM) was measured by using a CLARIOstar Plus microplate reader (BMG Labtech, Germany) to generate a standard curve. Concentration of dox loaded in ODNVs was then determined using the equation (f_O+D_-f_O_)/S where f_O+D_ and f_O_ are fluorescence intensities of dox-loaded ODNVs and unloaded ODNVs, respectively, and S the slope of the standard curve. The mode size and concentration of ODNVs before and after loading was determined by NTA.

For 2D culture, A549 and SKOV3 were seeded in 96 well plate at a density of 8000 cells/well, exposed to vehicle control, ODNVs (4.10^11^ ODNVs/mL), free dox at a concentration of 1 µM and ODNVs (4.10^11^ ODNVs/mL) loaded with dox at a total concentration of 1 µM for 3 days. The cell viability of each condition was evaluated by modified MTT as previously described.

For 3D culture, A549 and SKOV3 cells were seeded at a density of 4000 cells/well in 96-well Ultra-low Attachment Plates. After 4 days, spheroids were exposed to vehicle control, ODNVs (4.10^11^ ODNVs/mL), free dox at a concentration of 1 µM and ODNVs (4.10^11^ ODNVs/mL) loaded with dox at a total concentration of 1 µM. Spheroids were imaged using bright field microscopy every 2 days for 8 days and their long (D) and short (d) diameters measured using ImageJ. Spheroid volume (V) was then calculated by using the same formula as previously described.

### Statistics

Statistical analyses and graphical representations were performed and generated using GraphPad Prism 10.0.2 (GraphPad). For comparison of multiple groups, one-way ANOVA or two-way repeated measures ANOVA were used with Šídák’s multiple comparisons test applied when relevant for interpretation. For comparison of two groups, unpaired two-tailed Students *t*-tests were used. Differences to normalized control value were calculated using one sample t-test with a hypothetical value of 100. Data were evaluated for distribution patterns using tests including D’Agostino-Pearson and Shapiro-Wilk tests. Data represent mean ± s.e.m and the significance level for all tests was 0.05. Statistical significance is illustrated as follows: * *p* < 0.05; ** *p* < 0.01; *** *p* < 0.001, **** *p* < 0.0001. All experiments were performed from at least three independent batches or biological replicates.

## Results

### ODNVs physical and biochemical characterization

**Fig. 1:**
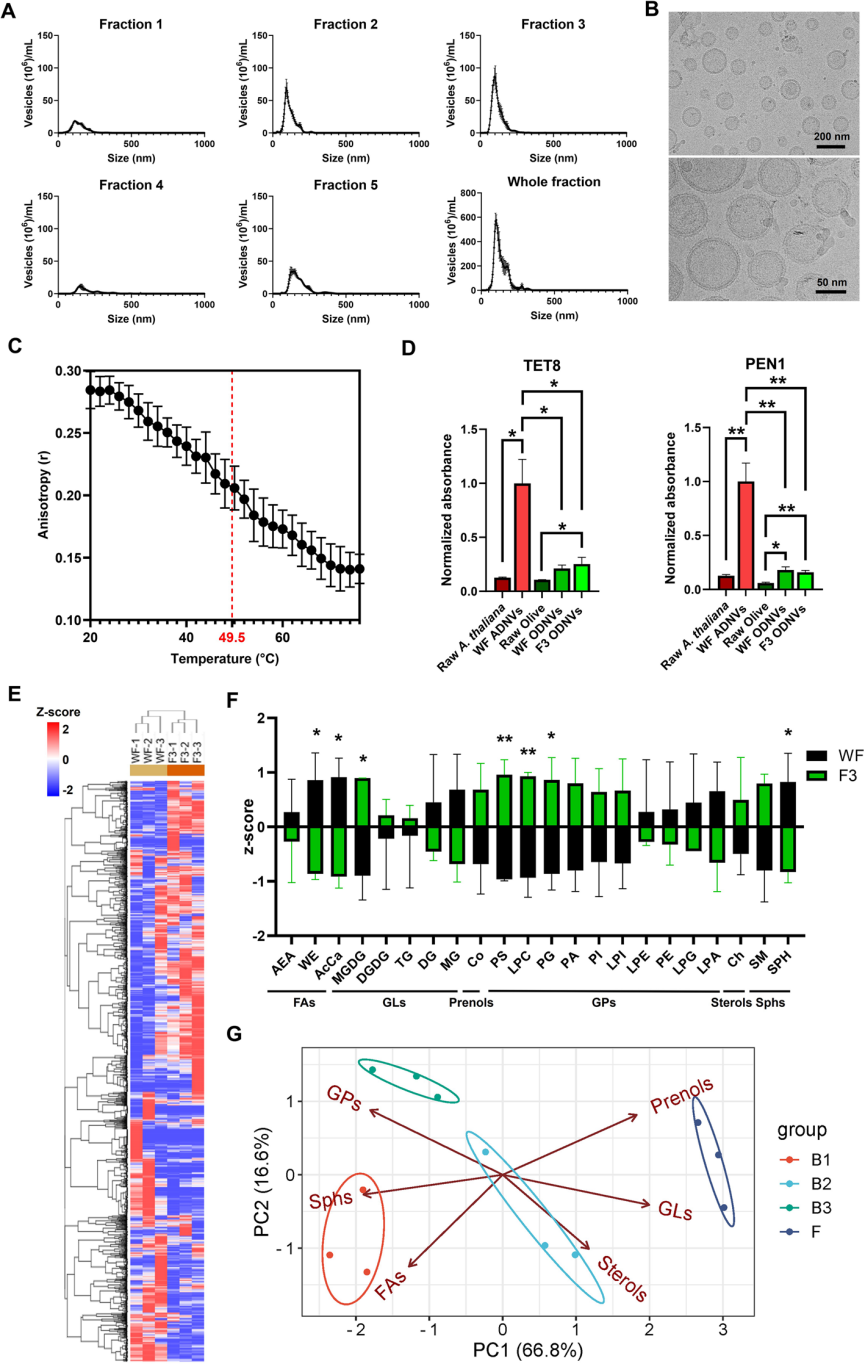
Characterization of ODNVs size, morphology and biochemical content. **A** Nanoparticle tracking analysis of size and quantity of the ODNVs collected in the different fractions from canned olives B1. **B** Representative cryo-EM images of F3 ODNVs. **C** ODNVs membrane fluidity at various temperatures (*n* = 3). Red dotted line indicates phase transition temperature. **D** Expression of TET8 and PEN1 proteins in the tissue and derived nanovesicles of *A thaliana* (ADNVs) and olive fruits (ODNVs). Data were normalized to protein level in *A. Thaliana* tissue (*n* = 3), and differences between each group were calculated using unpaired, two-tailed Student’s t-tests with a significance threshold of α < 0.05. **E** Hierarchical clustering of the 3240 common lipids identified in WF and F3. **F** Relative lipid abondance of the main lipid subclasses identified between WF and F3. Differences between each group were calculated using unpaired, two-tailed Student’s t-tests with a significance threshold of α < 0.05. **G** Principal component analysis showing expression of lipids of the 6 main lipid classes between 3 different brand of canned olives (B1, B2, and B3) and fresh, unprocessed olives (F). All data are represented as mean ± s.e.m. *p < 0.05; **p < 0.01; ***p < 0.001; ****p < 0.0001

ODNVs were isolated from canned black olives using differential centrifugations on blended olive fruits, followed by sucrose density gradient ultracentrifugation on the remaining supernatant (see Methods and Supplementary Fig. 1). In the whole fraction (WF), an average of 3.95.10^12^ total ODNVs could be isolated from 100 g of fruits using this protocol. Density gradient ultracentrifugation separated the WF in five distinct fractions (Fig. 1A). NTA revealed that all fractions had a PDI < 0.2 (the lowest 0.10 for fraction 2 and the highest 0.16 for fraction 4) showing a good uniformity of the size distribution suitable for DDS (Supplementary Table 1) [55]. However, fraction 3 (F3) contained the highest quantity of ODNVs (1.12.10^12^ ± 4.30.10^11^) compared to other fractions, and, with an average size of 109.5 ± 3.0 nm, was thus selected as working material for the rest of this study. First, F3 ODNVs were imaged by cryo-EM. Most of the particles had a round shape and were individual, although the overall population also displayed multilayer particles (where small vesicles were contained in a larger one) and particles with electron dense cargo in the lumen (Supplementary Fig. 3). Overall, cryo-EM images confirmed the ODNVs mean diameter of ~ 100 nm and, interestingly, showed the presence of a membrane bilayer with transmembrane proteins (Fig. 1B). To determine the charge of this membrane, electrophoretic mobility was then used and showed that, at neutral pH, the ODNVs zeta potential value was −20 ± 1 mV in 1 mM DPBS indicative of a negative membrane charge and suggesting a good colloidal stability. To study the physicochemical membrane properties of the ODNVs, the membrane fluidity was then analyzed at various temperatures and the phase transition temperature was revealed to be 49.5 °C ± 0.5 (Fig. 1C). Notably, ODNVs had a gradual increase of the membrane fluidity typical of membranes with rich composition compared to more simplistic models (e.g., liposomes). To identify the origin of these particles, the expression of tetraspanin-8 (TET8) and syntaxin PENETRATION1 (PEN1), two membrane proteins recently identified in *Arabidopsis thaliana* as plant EVs biomarkers was assessed [10, 56]. Results showed that the expression levels of TET8 and PEN1 proteins were, respectively, 2.5x and 2.7x higher in F3 ODNVs than in whole olive, highlighting an enrichment in ODNVs samples and suggesting an extracellular origin of these vesicles (Fig. 1D). Finally, lipidomics analysis was performed to assess the lipid composition of the different fractions as well as the possible variability between different sources. Analysis identified 3240 unique lipids (Supplementary Table 2) belonging to 6 main lipid classes (FAs, GLs, GPs, sterol and prenol lipids) and 42 subclasses (Supplementary Fig. 4A). Unsupervised hierarchical clustering analysis of WF and F3 lipids revealed that the two fractions separated into two distinct clusters, suggesting a difference in lipid composition (Fig. 1E). Although, all major lipid components in WF were clearly detected in F3, comparison of relative lipid abundance showed an enrichment in FAs for WF (WE, AcCa) while MGDG and GPs (PS, PG and LPG) were enriched in F3 (Fig. 1F). Analysis of the other fractions, F2 and F4, revealed a similar profile to F3 with hierarchical clustering showing one distant cluster for WF separated from one cluster regrouping all fractions (Supplementary Fig. 4B). F2 and F4 also showed identical lipid enrichment as F3 compared to WF with an increase in MGDG and GPs and decrease in FAs (Supplementary Fig. 4C-D). Finally, lipid composition of different sources of olives, including 3 different brands (B1, B2 and B3) of canned olives and a batch of fresh, unprocessed olives (F) were compared. NTA showed that B2 and B3 displayed similar profiles to B1 with an average ODNV diameter of 102.5 nm and 86.1 nm, respectively, and F3 being the fraction with the most monodispersed particle distribution and the highest concentration (Supplementary Fig. 5). Conversely, fresh olives showed a more heterogenous profile with lower ODNVs concentration, higher ODNVs size (average diameter = 114.5 nm) and fractions with polydispersed particle distributions. Principal component analysis showed a clear discrimination between each group, suggesting a distinct lipid composition for the 4 different sources (Fig. 1G). The fresh olives are mostly enriched in prenol lipids and GLs while FAs and Sphs are increased in B1 and GPs in B3. Moreover, comparison with animal EVs (average diameter = 95.7 nm) showed that ODNVs had lower content in sterols (Ch, ChE), SM, and lysoGPs (LPG, LPS) while ODNVs are enriched in GLs and FAs (Supplementary Fig. 6).

### ODNVs biocompatibility and cellular intake

**Fig. 2:**
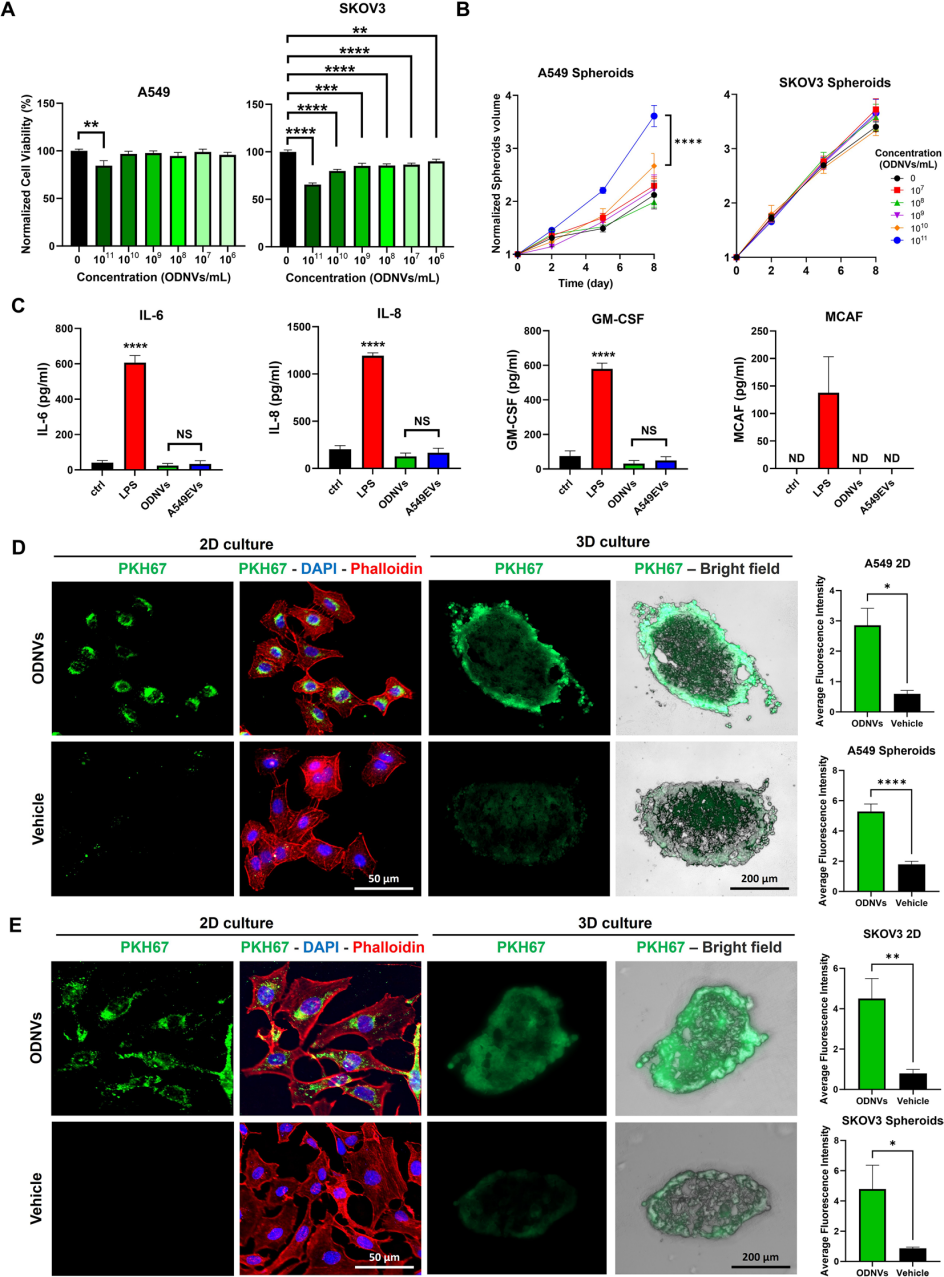
ODNVs cytotoxicity, immunogenicity and internalization in 2D and 3D culture of lung and ovarian cells. **A** Viability of A549 and SKOV3 cells cultured in 2D for 3 days and exposed to different concentration of ODNVs assessed by MTT (*n* = 3). Differences to unexposed sample were evaluated using unpaired, two-tailed Student’s t-tests with a significance threshold of α < 0.05. **B** Growth curves of A549 and SKOV3 spheroids exposed to different concentration of ODNVs for 8 days (*n* = 3). Data were analyzed using two-way repeated-measures ANOVA to assess the main effects of treatment. **C** Level of IL-6, IL-8, GM-CSF and MCAF released in HULEC-5a cells supernatant after exposure to 1.10^9^ A549 EVs/mL, 1.10^9^ ODNVs/mL and 1 µg/mL LPS for 24 h (*n* = 3). Differences to unexposed sample (ctrl), and between ODNVs and A549 EVs samples, were evaluated using unpaired, two-tailed Student’s t-tests with a significance threshold of α < 0.05. NS = Non-Significant; ND = Not Detected. Representative immunofluorescent pictures of A549 (**D**) and SKOV3 (**E**) cells cultured in 2D or 3D (spheroids) and incubated for 24 h with ODNVs (10^12^ ODNVs/mL) or PBS pre-stained with PKH67 green fluorescent cell membrane labeling. For 2D culture, cells were counterstained for DAPI (blue) and Alexa Fluor 555 Phalloidin (red) to stain DNA and F-actin and visualize nucleus and cytoplasm, respectively. Spheroids were embedded in OCT, sliced and imaged under bright field. Graphs represent the average fluorescence intensity of at least 100 cells or 3 spheroids from three independent experiments. Differences between samples incubated with PKH67-stained ODNVs or PBS were evaluated using unpaired, two-tailed Student’s t-tests with a significance threshold of α < 0.05. All data are represented as mean ± s.e.m. *p* < 0.05; **p < 0.01; ***p < 0.001; ****p < 0.0001

After demonstrating that ODNVs were double-layer lipid nanovesicles negatively charged, their interaction with cells was evaluated. In this objective, lung (A549) and ovarian (SKOV3) cancer cells were cultured both in two- (2D) and three- (3D) dimensional (as spheroids) systems and exposed to different concentrations of ODNVs. Results showed that, in 2D culture, viability of A549 cells exposed to different doses of ODNVs remained identical to control except for the cells exposed to the highest concentration (10^11^ ODNVs/mL) which showed a significant decreased viability of 15% (Fig. 2A). SKOV3 cells were revealed to be more sensitive to ODNVs exposure with a 10% decrease in viability at the lowest concentration (10^6^ ODNVs/mL) and up to 35% decrease at the highest concentration (Fig. 2A). In 3D culture, spheroids from both cell lines showed similar growth rates after ODNVs exposure for 8 days, at all concentration tested, compared to the unexposed control (Fig. 2B). Only A549 spheroids showed a significant increased volume after exposure to the highest ODNVs concentration.

In addition to cellular toxicity, the ODNVs immunogenicity was also evaluated by measuring the release of 8 pro-inflammatory cytokines by normal endothelial cells (HULEC-5a) after exposure to a concentration of 1.10^9^ ODNVs/mL, 1.10^9^ A549 EVs/mL and LPS (positive control). Although IL-1α, IL-1β, IFN-γ and TNF-α were not detected, results showed that levels of IL-6, IL-8, GM-CSF and MCAF were increased after cells were exposed to LPS while their level remained unchanged after ODNVs exposure compared to both control and A549 EVs (Fig. 2C), suggesting that ODNVs, like A549 EVs, did not elicit immune response.

Then, to assess cellular intake, ODNVs were labeled with a PKH67 fluorescent membrane stain and incubated for 24 h with A549 and SKOV3 2D and 3D cultures. Fluorescence microscopy revealed that ODNVs were efficiently internalized in the cytoplasm of both cell lines (Fig. 2D-E). For A549 cells, fluorescence intensity in ODNVs-exposed cells was 4.8x and 2.9x significantly higher than unexposed cells in 2D and 3D systems, respectively. For SKOV3, fluorescence intensity in ODNVs-exposed cells was 5.7x and 5.5x significantly higher than unexposed cells in 2D and 3D systems, respectively.

### ODNVs resistance to thermal, osmotic, pH and mechanical stress

**Fig. 3:**
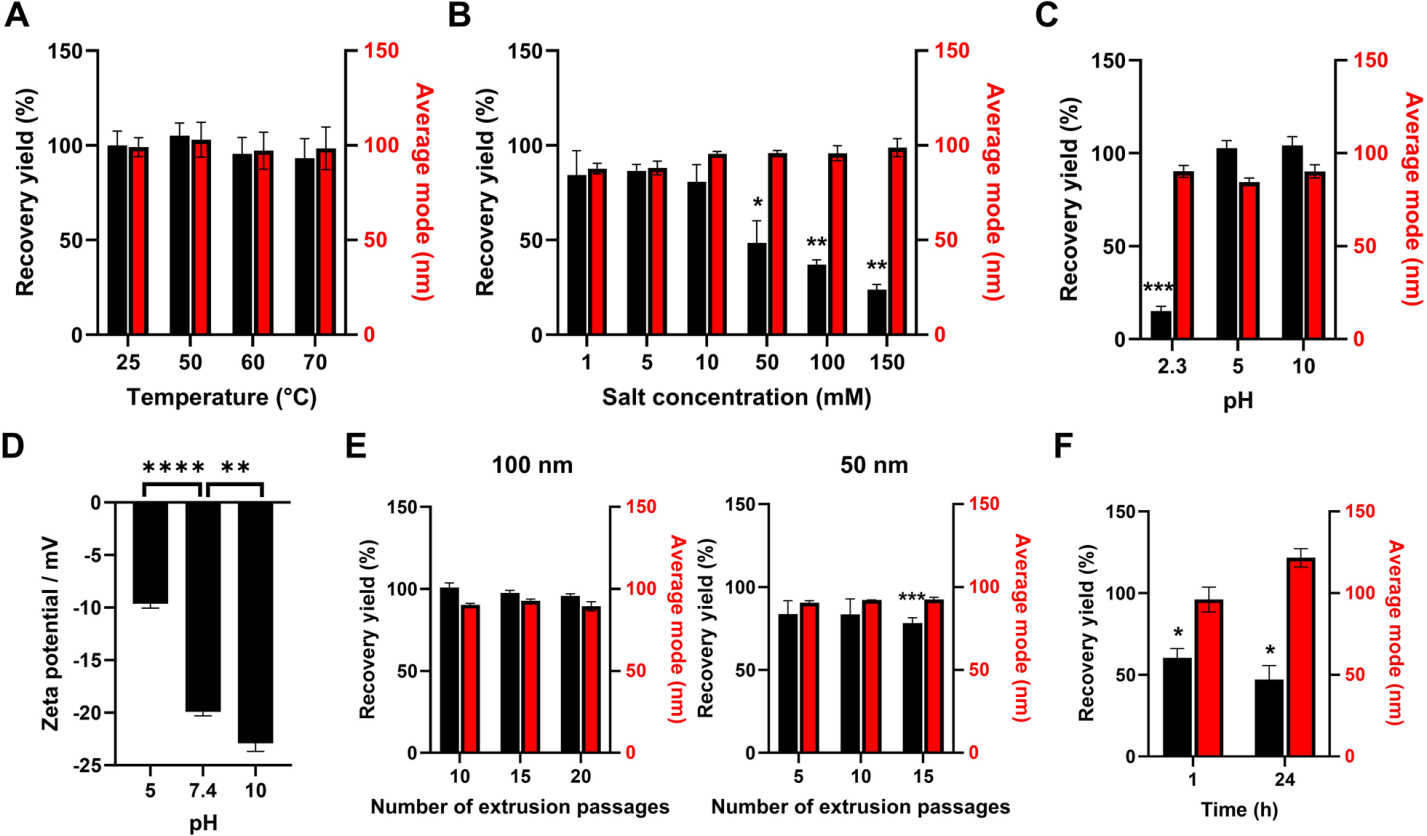
ODNVs resistance to heat, salt, pH, mechanical deformation and blood-like environment. **A** Relative quantity and mode of ODNVs after 1 h incubation in water at different temperature and compared to ODNVs stored at 4 °C (*n* = 3). **B** Relative quantity and mode of ODNVs after 24 h incubation at 4 °C in solutions with different salt concentration and compared to solution with no salt (*n* = 3). **C** Relative quantity and mode of ODNVs after 24 h incubation at 4 °C in aqueous solutions of different pH and compared to solution of pH = 7.4 (*n* = 3). Differences to pH 7.4 were evaluated using unpaired, two-tailed Student’s t-tests with a significance threshold of α < 0.05. **D** Zeta potential of ODNVs after 24 h incubation at 4 °C in aqueous solutions (1 mM salt) of different pH (*n* = 3). **E** Relative quantity and mode of ODNVs subjected to serial extrusion through 100 and 50 nm porous membrane and compared to native ODNVs (*n* = 4). **F** Relative quantity and mode of ODNVs after 1 h and 24 h incubation at 37 °C in exosome depleted serum (*n* = 3). All conditions were tested at 1.10^9^ ODNVs/mL. All data are represented as mean ± s.e.m. Differences were evaluated using one sample t-test with a hypothetical value of 100: *p < 0.05; **p < 0.01; ***p < 0.001; ****p < 0.0001

An important feature of lipid nanoparticle delivery systems is their resistance to external stresses that can provide improved stability and offer additional approaches to enhance therapeutic loading. Therefore, to evaluate ODNVs resistance to environmental constraints, a series of experiments was performed where ODNVs recovery yield and size were measured after exposure to different external stresses. First, ODNVs incubation in water bath heated up to 70 °C did not trigger any decrease in recovery yield or changes in ODNVs size (Fig. 3A). Second, ODNVs also showed good resistance to an incubation with a increased salt concentration up to 10 mM, with a recovery yield and size unchanged compared to an incubation without any salt (Fig. 3B). However, for higher salt concentrations, although ODNVs size was not affected, the recovery yield significantly decreased to 48%, 37% and 24% after incubation with salt concentration of 50, 100 and 150 mM, respectively. Third, ODNVs were also incubated in aqueous solutions at different pH. Our data showed that incubation in solutions at pH 5 or 10 did not affect the ODNVs recovery yield and size compared to an incubation in a solution at pH 7.4 (Fig. 3C). However, at pH 2.3, although their size stayed unchanged, the recovery yield of ODNVs drastically decreased to 15%. As pH has previously been shown to affect zeta potential [57], the ODNVs surface charge at pH 5 and 10 was then measured. Results showed that increasing pH significantly decreased zeta potential (−23 ± 2 mV at pH 10) while decreasing pH significantly increased zeta potential (−10 mV ± 1 at pH 5) compared to the zeta potential of −20 ± 1 mV previously measured at pH 7.4 (Fig. 3D). ODNVs deformability looking at their resistance to extrusion was also assessed by passing them through membranes with pore size of 50–100 nm. Our data indicated that serial passages, up to 20, through 100 nm pores membranes did not affect ODNVs recovery yield or size (Fig. 3E). Even passages through smaller pores (50 nm) did not change ODNVs size compared to fresh ODNVs, with only a small, but significant, decrease of 22% in recovery yield for ODNVs up to 15 passages. Finally, to evaluate their stability in a blood-like environment, ODNVs were incubated in 50% exosome depleted serum at 37 °C for 1 and 24 h. Our data showed that the ODNVs recovery yield significantly decreased by 40% and 53% after 1 and 24 h incubation respectively, while ODNVs mode size remained unchanged.

### Assessment of ODNVs optimal storage conditions

**Fig. 4:**
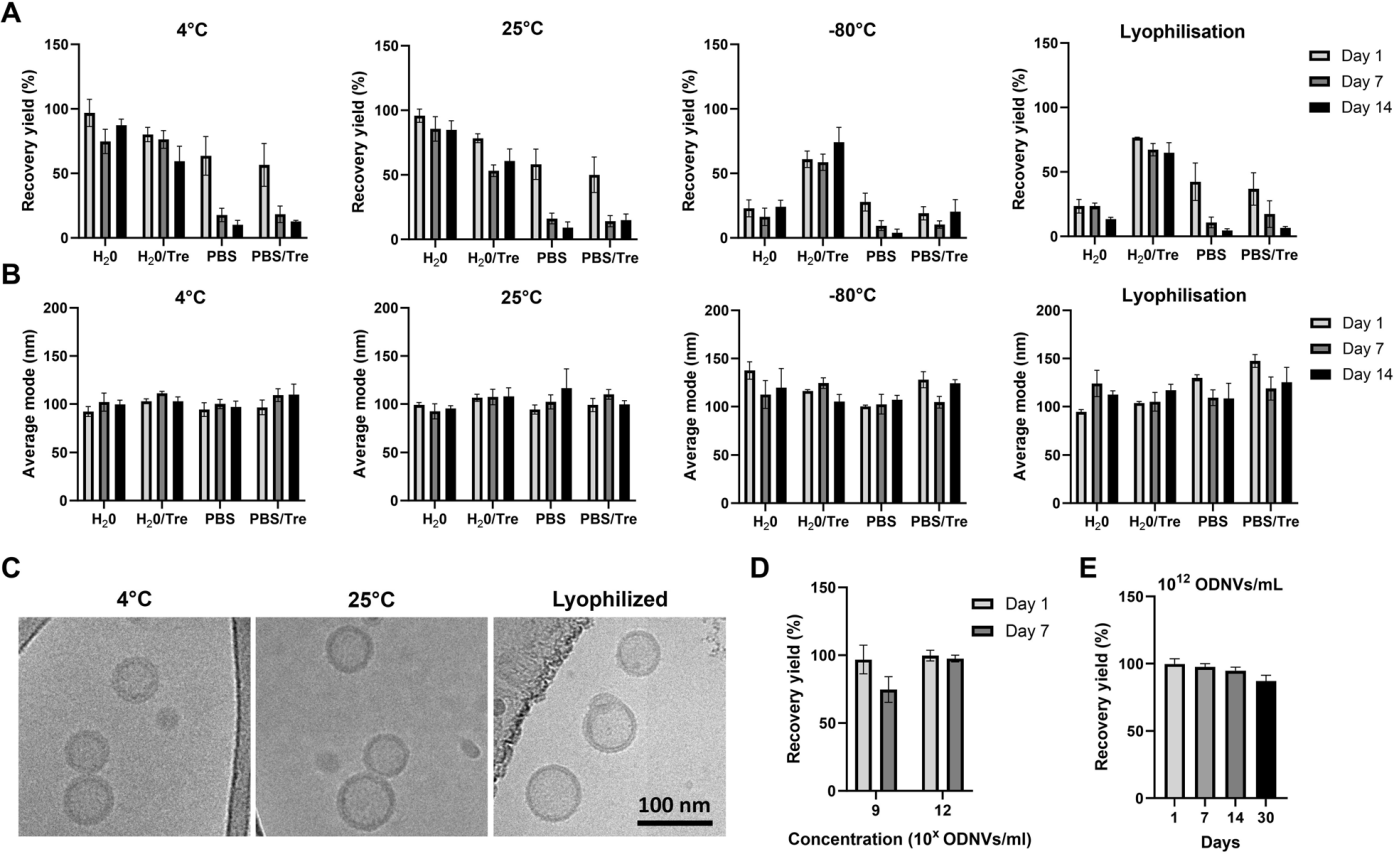
ODNVs size, yield and structure under different storage conditions. Recovery yield (**A**) and average size (**B**) of ODNVs (10^9^ ODNVs/mL) under different storage conditions. Samples were resuspended in different storage buffers including DI water (H_2_O), water + trehalose (H_2_O/Tre), PBS and PBS + trehalose (PBS/Tre), and kept at refrigerated (4 °C), room (25 °C), freezing (-80 °C) temperature or lyophilized. After 24 h, frozen and lyophilized samples were thawed and reconstituted in water at 4 °C. All samples were then stored for 1, 7 and 14 days. All conditions were compared to fresh ODNVs (*n* = 3). **C** Cryo-EM pictures of ODNVs stored for 14 days at 4 °C, 25 °C in H_2_O and in lyophilized condition after resuspension in H_2_O/Tre. **D** Recovery yield of ODNVs stored at different concentrations for 7 days at refrigerated temperature in water and compared to fresh ODNVs (*n* = 3). **E** Recovery yield of ODNVs stored at high concentration (10^12^ ODNVs/mL) up to 1 month at refrigerated temperature in water and compared to fresh ODNVs (*n* = 3). All data are represented as mean ± s.e.m

Based on this data that demonstrated good resistance to multiple stresses, the ODNVs were then challenged with various storage conditions in order to determine the minimum requirements for their long-term preservation. Therefore, 1.10^9^ ODNVs/mL were stored at refrigerated (4 °C), ambient (25 °C) and freezing (−80 °C) temperatures, as well as after lyophilization, in different buffers including water and PBS, supplemented or not with trehalose at 25 mM [58]. Frozen and lyophilized samples were thawed and reconstituted in DI H_2_O at 4 °C. After recovery, all samples were stored for 1, 7 or 14 days. Interestingly, data showed that on day 14, the highest recovery yields were obtained after storage at refrigerated or ambient temperature in water with only a reduction of 13 and 15%, respectively, compared to fresh ODNVs (Fig. 4A). The addition of trehalose to water did not improve the recovery yield in these conditions. For storage at freezing temperature or under lyophilized conditions, water with trehalose buffer provided the highest recovery yield at day 14 (74% and 65%, respectively). Finally, storage in PBS, with and without trehalose, at any temperature, greatly decreased ODNVs recovery yield and proved to be inefficient as storage buffer. This result was consistent with our previous data demonstrating the negative impact of salt concentration on ODNVs recovery yield (Fig. 3B). To note, none of these challenges was shown to significantly affect the ODNVs size (Fig. 4B). Moreover, cryo-EM pictures confirmed that storage in water at 4 °C and 25 °C or in lyophilized condition in water with trehalose for 14 days did not alter the ODNVs structure (Fig. 4C). Interestingly, it was also noticed that the ODNVs concentration affected the storage efficiency. Indeed, recovery yield of ODNVs stored at a concentration of 10^12^ ODNVs/mL at 4 °C in water increased from 75 to 97.5% on day 7 compared to ODNVs stored at a lower concentration (10^9^ ODNVs/mL) (Fig. 4D). At high concentration, ODNVs recovery yield stayed stable for up to 1 month (Fig. 4E).

### ODNVs drug loading and release

**Fig. 5:**
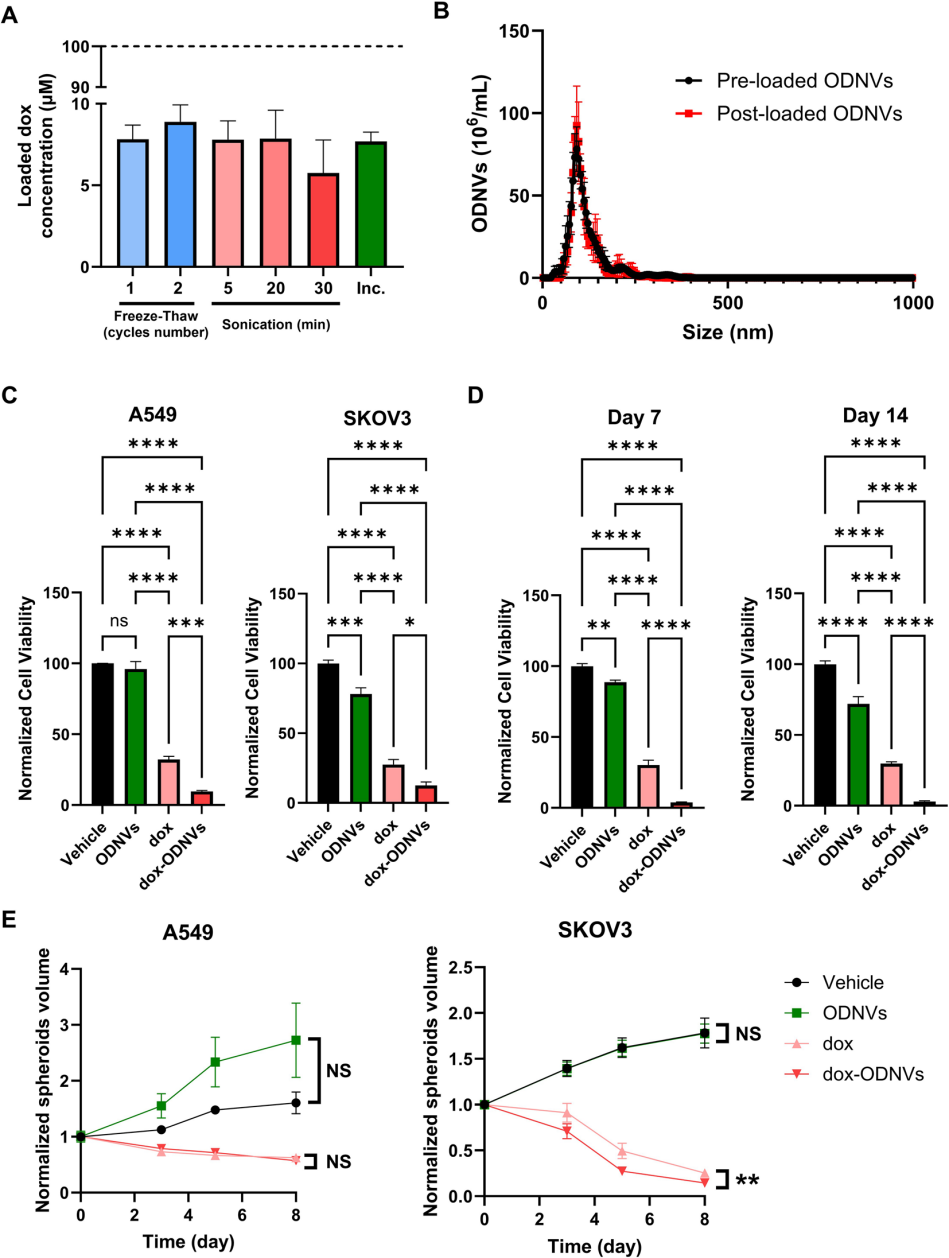
Chemotherapeutic drug delivery to 2D and 3D lung and ovarian cancer cells using ODNVs. **A** Concentration of doxorubicin (dox) loaded in ODNVs by 1 or 2 freeze-thaw cycles, sonication for 5, 20 and 30 min and passive incubation (Inc.) for 4 h at 37 °C (*n* = 3). Initial dox concentration was 100 µM (dotted line). **B** Nanoparticle tracking analysis of mode and quantity of the ODNVs before and after loading with doxorubicin (100 µM) by passive incubation at 37 °C (4 h). **C** Viability of A549 and SKOV3 cells exposed to vehicle (water), ODNVs (4.10^11^ ODNVs/mL), free doxorubicin (dox) at a concentration of 1 µM and ODNVs (4.10^11^ ODNVs/mL) loaded with dox (dox-ODNVs) at a total concentration of 1 µM for 3 days (*n* = 3). Differences were evaluated using one-way ANOVA with Tukey’s multiple comparisons test. **D** Viability of A549 cells exposed to vehicle (water), ODNVs (4.10^11^ ODNVs/mL), free doxorubicin (dox) at a concentration of 1 µM and ODNVs (4.10^11^ ODNVs/mL) loaded with dox (dox-ODNVs) at a total concentration of 1 µM for 3 days (*n* = 3). ODNVs and dox-loaded ODNVs were previously stored at 4 °C in water for 7 and 14 days. Differences were evaluated using one-way ANOVA with Tukey’s multiple comparisons test. **E** Volume growth of A549 and SKOV3 spheroids exposed to vehicle (water), ODNVs (4.10^11^ ODNVs/mL), free doxorubicin (dox) at a concentration of 1 µM and ODNVs (4.10^11^ ODNVs/mL) loaded with dox (dox-ODNVs) at a total concentration of 1 µM for 8 days (*n* = 3). Dataset were plotted using the second order polynomial model and differences were calculated using extra sum-of-squares F test. All data are represented as mean ± s.e.m: *p < 0.05; **p < 0.01; ***p < 0.001; ****p < 0.0001

Finally, to demonstrate whether ODNVs could serve as a DDS, their ability to load and release a bioactive drug was assessed. First, different approaches were explored to load the hydrophilic anticancer drug doxorubicin-HCl (dox). ODNVs were exposed to 100 µM dox, treated by freeze-thaw method, sonication, or passive incubation, and then filtered to remove unloaded dox. Results showed that all these protocols allowed dox loading with a similar efficacy of ~ 8% (Fig. 5A). Therefore, the simple protocol of passive incubation was selected as a method of choice for dox loading. NTA showed that the number (4.8.10^12 ^vs. 4.5.10^12^ ODNVs/mL) and average mean (92.5 nm) of ODNVs were similar pre- and post-loading suggesting that the treatment did not modify the size and concentration of the ODNVs (Fig. 5B). To determine if the ODNVs could then release dox and induce a biological response, A549 and SKOV3 cells were exposed to 1 µM of free dox and ODNVs-loaded dox. Our data showed that in A549 and SKOV3 cells, treatment with dox decreased cell viability to 32% and 28% respectively (Fig. 5C). Interestingly, when dox was delivered by ODNVs, cell viability was significantly lower than with free dox (10% and 13% for A549 and SKOV3 cells, respectively), suggesting that ODNVs delivery enhanced dox cytotoxicity effect. Importantly, this differential effect was also assessed after dox-loaded ODNVs were stored for 7 and 14 days at 4 °C in water (Fig. 4A). Data demonstrated that cell viability of A549 cells was 4% and 3% after exposure to dox-loaded ODNVs stored for 7 and 14 days respectively, while exposure to the same concentration of free dox (1 µM) reduced cell viability to only 30% compared to vehicle (Fig. 5D). These results confirmed that dox triggered a higher cytotoxicity when loaded in ODNVs than delivered in free form and that storage conditions up to 14 days did not compromise the efficiency of the dox. However, it is worth noting that viability of cells exposed to dox-loaded ODNVs stored for 7 and 14 days (Fig. 5D) was lower than after exposure to freshly prepared dox-loaded ODNVs (Fig. 5C). This result must be placed in parallel to the decreased viability of cells exposed to ODNVs alone that have been stored for 7 and 14 days (89% and 72%, respectively, compared to vehicle), suggesting an intrinsic ODNVs cytotoxicity after long-term storage in these conditions (Fig. 5D). Finally, A549 and SKOV3 spheroids were also exposed to free and ODNVs-loaded dox and their growth monitored for 8 days. For both cell lines, results showed that dox treatment, either delivered freely or loaded in ODNVs, drastically reduced spheroids volume over time (Fig. 5E). Although no significant differences were visible for A549 spheroids, SKOV3 spheroids growth rate was significantly lower with dox delivered in ODNVs than free dox directly in the medium.

## Discussion

To evaluate the potential of olives to provide natural nanocarriers and serve as DDS, it was first demonstrated that using serial centrifugation, lipid nanovesicles could be isolated from olive fruits with a high yield of ~ 4.10^10^ particles/g tissue, similar to yield usually obtained from plants [21]. The average diameter of these ODNVs obtained from the purest fraction was ~ 110 nm, consistent with the size of most of PDNVs extracted so far [20] and falling in the range of animal exosomes [59]. The enrichment of these ODNVs in TET8 and PEN1 suggested an extracellular origin. However, the expression level of these proteins in ODNVs was much lower than *A. Thaliana*-derived nanovesicles. TET8 and PEN1 being biomarkers of two distinct subclasses of plant EVs [60], this could suggest that TET8 and PEN1 expression is tissue-dependent and that ODNVs could carry different biomarkers or originate from another biogenesis pathways. The lipidomics analysis revealed a wide diversity of lipids present in ODNVs, which, along with membrane fluidity assessment and microscopy imaging, suggested a complex composition that would need to be characterized via comprehensive (multi)omics studies from a drug delivery perspective [5]. Moreover, an important consideration in nanovesicle manufacturing for drug delivery applications is that EVs generated from different sources showed important variability and biological response. Therefore, the lipidomics profiles of olives from different providers were compared and showed distinct differences between each batch, highlighting variability in lipid composition. The olives used in this study were canned black pitted olives and, therefore, underwent a manufacturing process that could have altered this composition. However, the physicochemical and biological properties have not been compared and further studies would be required to understand if this difference in lipid composition alter their function, and if the features measured in this study are common to all olive species or dependent on certain treatment. Overall, the average size, low polydispersity index, negative charge and lipidic composition of ODNVs suggested that they could be efficiently internalized by cells. Our data confirmed cellular uptake, however, the mechanism of internalization has not been investigated in the present work. Studies suggested that particles with a diameter < 200 nm involved clathrin-coated pits [55, 61] but the size dependance of this internalization is also affected by the surface charge [62], and additional studies would be required to better understand the biological properties of ODNVs and their interaction with cells. Interestingly, our results also suggested that ODNVs did not trigger the release of pro-inflammatory cytokines suggesting that ODNVs do not cause an inflammatory response and confirmed data from numerous previous studies showing the low immunogenicity of vesicles derived from edible plants [19, 22, 63].

As a nanocarrier, the ODNVs must resist various environmental stress to protect and deliver the loaded cargo. Therefore, their stability to multiple physical and chemical conditions was evaluated and revealed their high resistance to different stress factors. Results showed stable size and recovery yield of ODNVs at temperatures up to 70 °C, at different pH levels from 5 to 10 and even after mechanical stress deformation. These properties are critical for an efficient drug delivery system as they can offer good tolerance to the multi-step process required for the scale-up and manufacturing, including drug loading, purification, harvesting or engineering [5]. It also directly impacts the storage, a critical step in the handling of EVs: high resistance to ambient environmental conditions could minimize the heavy logistic usually involved in the preservation of nanovesicles [5]. There is still limited understanding on PDNVs stability, and, therefore, most of data originate from studies interrogating animal EVs [64]. Indeed, animal EVs have shown to be sensitive to changes in their environment with, for instance, a decrease in particle concentration as pH varied [65] or as temperature increased [66]. Studies have shown that PBS is the standard choice for resuspension, and the current consensus seems to support storage at − 80 °C [67] while storage at 4 °C or room temperature causes vesicles damage, aggregation and reduction in size and concentration [68–70]. However, these data must be taken with caution as contradictory results have shown that a one month storage at 4 °C did not affect exosome concentration [71] while − 80 °C storage drastically decreased the number of particles [72]. For plants, one study showed the good stability of black ginger nanovesicles at 4 °C and even under high acid pH (1.2) [73]. Another study demonstrated that storage temperatures altered the stability, size distribution, protein content, surface charge, and cellular uptake of *Dendropanax morbifera* leaf-derived nanovesicles and that with the appropriate buffer, a stable preservation could be obtained at 4 °C for 1 month [74]. Blueberry-derived nanovesicles have also been shown to be more stable at 4 °C and − 80 °C in PBS for short- and long-term storage, respectively, although some changes in size and decrease in protein concentration could still be observed [75]. In this work, it was demonstrated that ODNVs are stable in size and concentration for up to one month at 4 °C in water and not in PBS. These results were consistent with our data showing that ODNVs yield decreased with an increased salt concentration (Fig. 3B), suggesting a low osmolarity of ODNVs (~ 10 mOsm/L). This observation was also in line with the characterization of ODNVs stability in a blood-like environment whose osmolarity was identical to PBS and human blood (~ 300 mOsm/L) and that showed that recovery yield decreased by 50% after 24 h incubation (Fig. 3F). In vivo studies would be required to assess the ODNVs clearance in blood, but this preliminary in vitro data suggested that ODNVs would stay sufficiently stable before clearance, pharmacokinetics of lipid nanoparticles having shown a decrease > 95% in particles concentration in blood 24 h post-injection [76–78].

It was also showed that ODNVs concentration affected preservation quality, with a high concentration in ODNVs leading to a higher recovery yield. Such observation could explain the discrepancy often seen in the literature and emphasize the need to standardize the storage procedure guidelines. The origin of EVs could also highly impact their behavior and explain their difference in physical resistance under different environmental conditions. This is particularly relevant for plant kingdom that offers heterogenous sources that could generate EVs with diverse properties. In addition, the method of extraction from plants, where EVs can be both directly obtained from the apoplast or isolated after a total tissue disruption, could also generate EVs with different features. Our study mostly assessed physical integrity of ODNVs (obtained after tissue disruption) and did not perform extensive investigation on their biological properties and bioavailability under these different conditions. However, it was also proven that ODNVs loaded with a bioactive drug and stored in minimal conditions had the same efficiency as freshly loaded ODNVs. This demonstrated that the drug release was not affected by storage and suggested that the ODNVs biochemical properties and cellular uptake were not widely changed by the 4 °C storage.

Several studies have shown that PDNVs could be used to load exogeneous cargos, such as chemotherapeutic drugs [27, 79], miRNA [43, 80], siRNA [17] and even mRNA [81]. Our data demonstrated that ODNVs could load and deliver a chemotherapeutic drug which then showed higher cytotoxicity than when delivered unencapsulated. Interestingly, this result could be obtained using native, untransformed, ODNVs and a passive loading method. This contrasts with other plant-derived vesicles that may require lipid extraction and reassembly into nano sized particles for efficient loading [17]. Nevertheless, the high resistance of ODNVs to many physical and chemical constraints potentially allows alternative loading approaches that have not been assessed in this study (e.g., extrusion, electroporation, chemical treatment). Such approaches could increase the loading efficiency to ultimately limit the total amount of required ODNVs and thus decrease the cytotoxicity sometimes observed on certain cell types.

## Conclusion

This work demonstrated that lipid nanovesicles can be produced from olive fruits. These ODNVs were nanosized and negatively charged, had a low PDI, were made of a rich lipid bilayer, were biocompatible, had low immunogenicity, and could be internalized in 2D and 3D cellular structures. Interestingly, these ODNVs also showed high resistance to physical and chemical conditions, including high temperatures, pH, and mechanical deformations. This high resistance allowed them to be stored in minimal environment for a long period (4 °C in water for 1 month). Finally, ODNVs were able to load and release a chemotherapeutic drug that led to an increased efficacy compared to unencapsulated drug. In summary, these properties suggested that ODNVs are a promising drug delivery system candidate that could be used in many applications, including for routes of administration with harsh environmental conditions or for engineering modifications to enhance their functions.

## Supplementary Information

Supplementary Material 1.

Supplementary Material 2.

Supplementary Material 3.

## Data Availability

The datasets supporting the conclusions of this article are included within the article and its additional files.
