# Supplementary material for: Physical, biochemical, and biological characterization of olive-derived lipid nanovesicles for drug delivery applications
Source: J Nanobiotechnology. 2024 Nov 18;22:720. doi: 10.1186/s12951-024-02964-w (PMC11575425; doi:10.1186/s12951-024-02964-w)
Supplement: Supplementary file 3 — Supplementary Material 3. [file 12951_2024_2964_MOESM3_ESM.pdf]

Supplementary data

Physical, biochemical, and biological characterization of olive-derived lipid nanovesicles for drug delivery applications

Figures

Supplementary Figure 1: Workflow and timeline of the isolation of the olive-derived nanovesicles (ODNVs). Created with BioRender.com.
Supplementary Figure 2: Nanoparticle tracking analysis of size and quantity of the A549 EVs
Supplementary Figure 3: Cryo-EM images of ODNVs. **A**. Double vesicles **B.** Multivesicles **C.** Vesicles with electron dense cargo in lumen. Scale bars = 25 nm
Supplementary Figure 4: **A.** Alluvial diagram showing the 6 main classes and 42 subclasses of lipids identified in ODNVs. **B.** Hierarchical clustering of the 3240 common lipids identified in WF and fraction F2, F3 and F4. Relative lipid abondance of the main lipid subclasses identified between WF and F2 (**C**) and F4 (**D**). Differences between each group were calculated using unpaired, two-tailed Student's t-tests with a significance threshold of $\alpha<0.05$. All data are represented as mean ± s.e.m. p<0.05; **p<0.01; ***p<0.001; ****p<0.0001.
Supplementary Figure 5: Nanoparticle tracking analysis of size and quantity of the ODNVs collected in the different fractions from canned olives brand 2 (B2) (**A**), brand 3 (B3) (**B**) and fresh, unprocessed olives (**C**).
Supplementary Figure 6: **A.** Hierarchical clustering of the 3240 common lipids identified in WF of canned olives from brand 1 (B1), brand 2 (B2), brand 3 (B3), WF of fresh, unprocessed olives (F) and WF of animal EVs (A). **B.** Relative lipid abondance of the main lipid subclasses identified in B1, B2, B3, F and A samples.

Tables

Supplementary Table 1: Total number of particles and size of mode (nm) for each of the ODNVs fraction recovered after sucrose gradient.
Supplementary Table 2: List of identified lipids in ODNVs from brand 1 WF (sample A), F2 (sample B), F3 (sample C), F4 (sample D); brand 2 WF (sample E); brand 3 WF (sample F); fresh WF (sample G) and animal EVs WF (sample H).

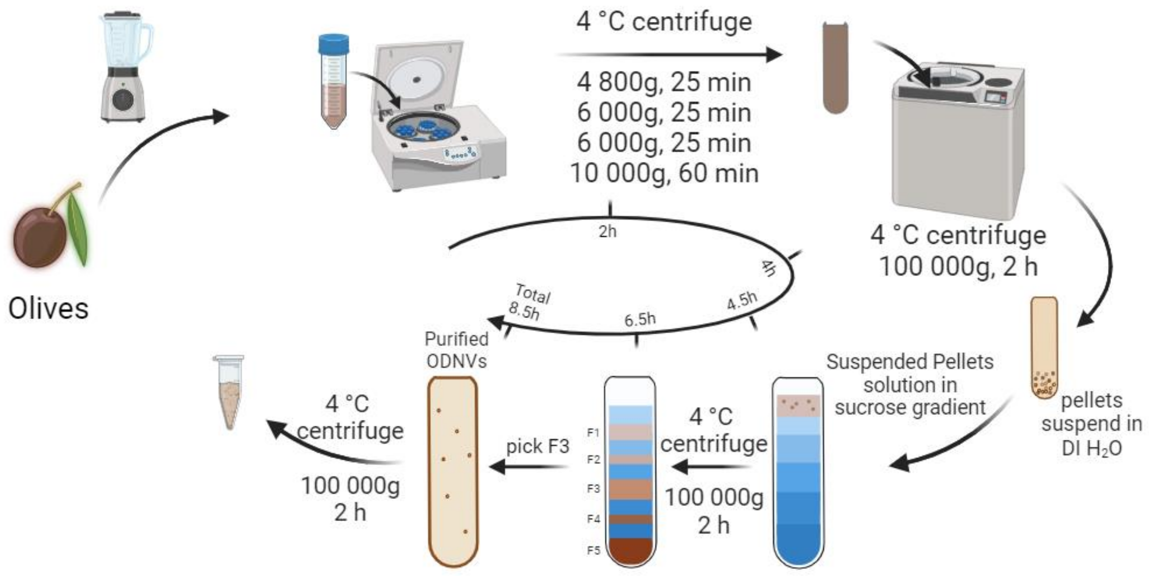

Supplementary Figure 1: Workflow and timeline of the isolation of the olive-derived nanovesicles (ODNVs). Created with BioRender.com.

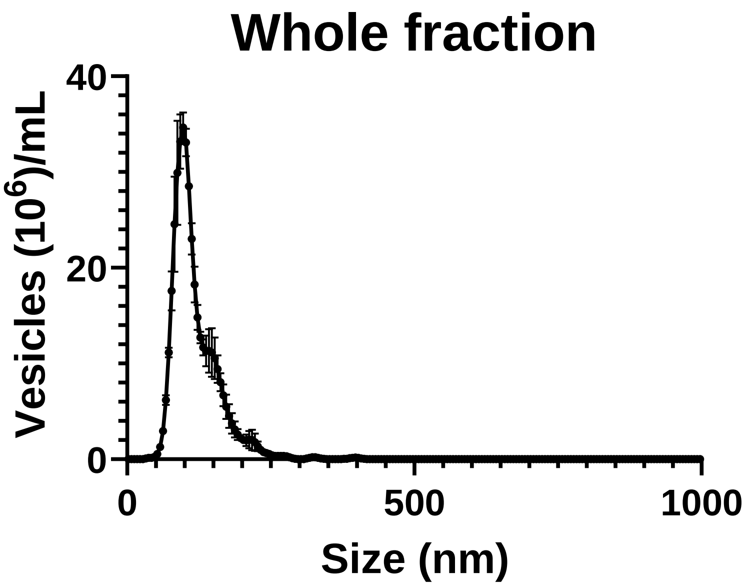

Supplementary Figure 2: Nanoparticle tracking analysis of size and quantity of the A549 EVs whole fraction

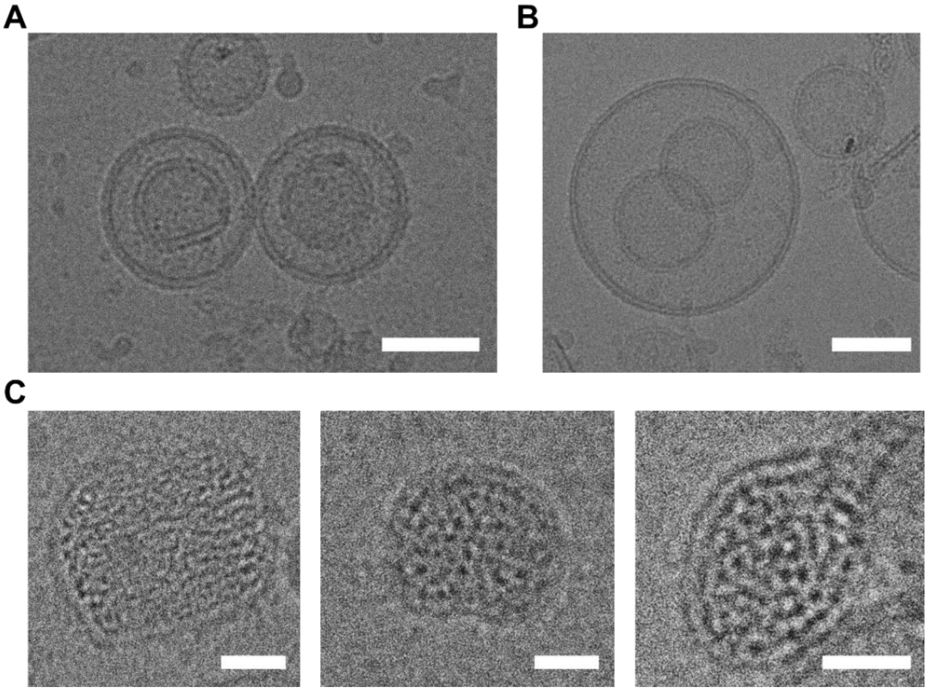

Supplementary Figure 3: Cryo-EM images of ODNVs. **A**. Double vesicles **B.** Multivesicles **C.** Vesicles with electron dense cargo in lumen. Scale bars = 25 nm

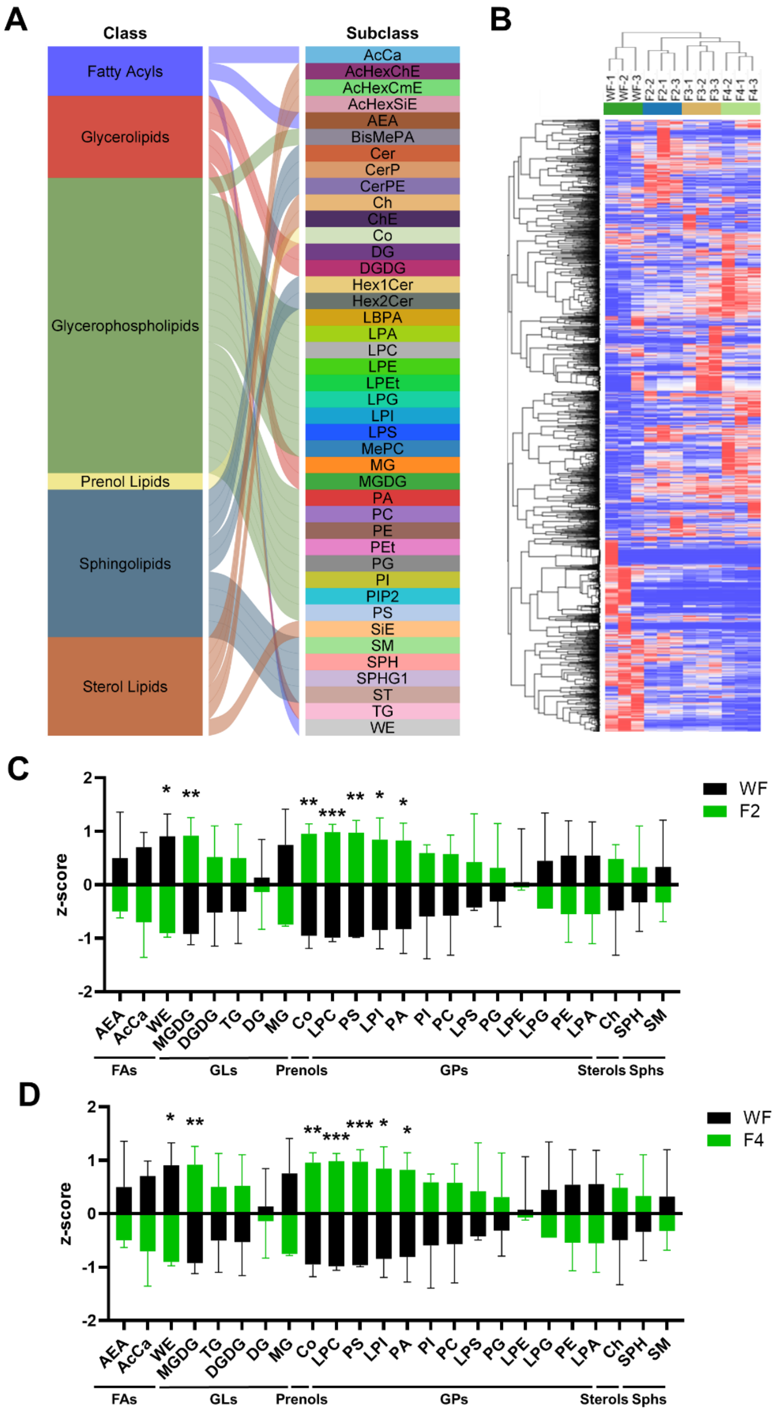

Supplementary Figure 4: **A.** Alluvial diagram showing the 6 main classes and 42 subclasses of lipids identified in ODNVs. **B.** Hierarchical clustering of the 3240 common lipids identified in WF and fraction F2, F3 and F4. Relative lipid abondance of the main lipid subclasses identified between WF and F2 (**C**) and F4 (**D**). Differences between each group were calculated using unpaired, two-tailed Student's t-tests with a significance threshold of α<0.05. All data are represented as mean ± s.e.m. p<0.05; **p<0.01; ***p<0.001; ****p<0.0001.

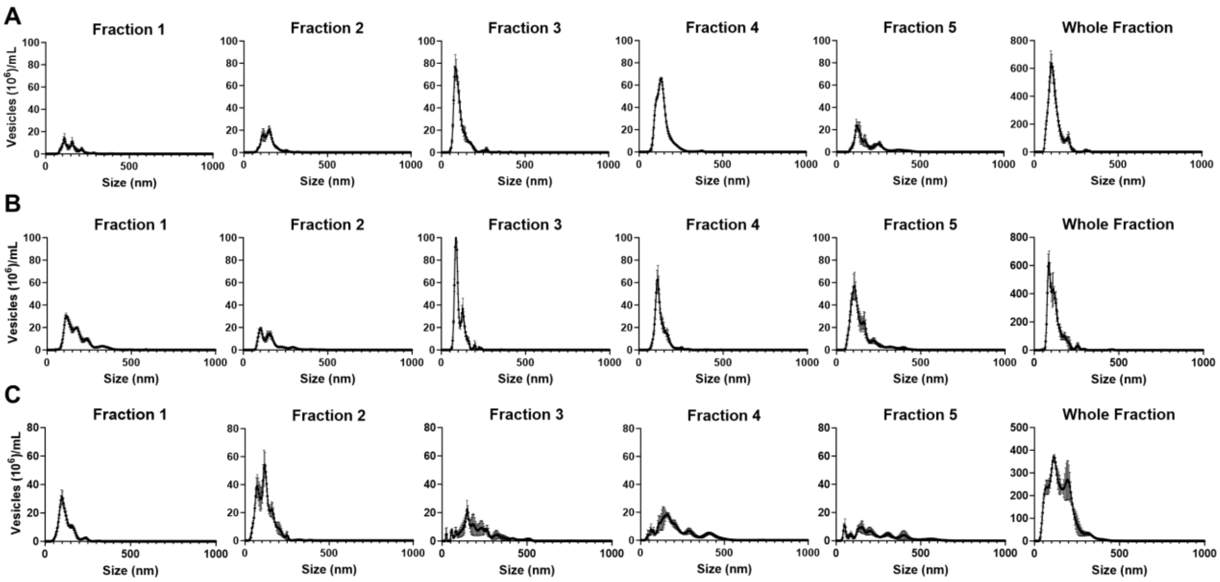

Supplementary Figure 5: Nanoparticle tracking analysis of size and quantity of the ODNVs collected in the different fractions from canned olives brand 2 (B2) (**A**), brand 3 (B3) (**B**) and fresh, unprocessed olives (**C**).

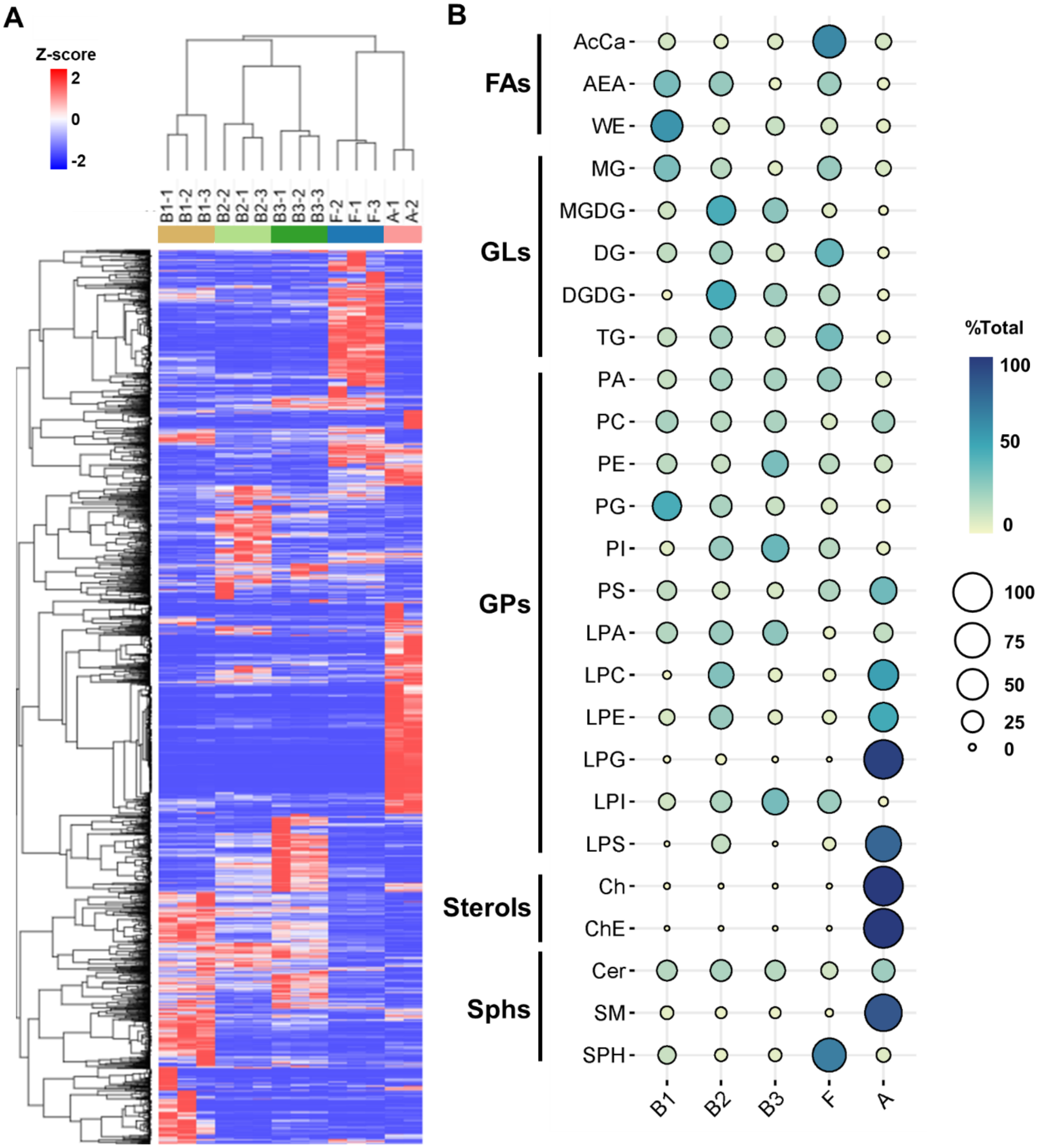

Supplementary Figure 6: **A.** Hierarchical clustering of the 3240 common lipids identified in WF of canned olives from brand 1 (B1), brand 2 (B2), brand 3 (B3), WF of fresh, unprocessed olives (F) and WF of animal EVs (A). **B.** Relative lipid abondance of the main lipid subclasses identified in B1, B2, B3, F and A samples.
